# Analysis of the Masked Metabolite of Deoxynivalenol and Fusarium Resistance in CIMMYT Wheat Germplasm

**DOI:** 10.3390/toxins9080238

**Published:** 2017-07-29

**Authors:** Hiroyuki Nakagawa, Xinyao He, Yosuke Matsuo, Pawan K. Singh, Masayo Kushiro

**Affiliations:** 1 Food Research Institute, National Agriculture and Food Research Organization (NARO), 2-1-12 Kannon-dai, Tsukuba-shi, Ibaraki 305-8642, Japan; hironkgw@affrc.go.jp (H.N.); ymatuo@affrc.go.jp (Y.M.); 2 Advanced Analysis Center, NARO, 2-1-12 Kannondai, Tsukuba 305-8642, Japan; 3 International Maize and Wheat Improvement Center (CIMMYT), Apdo, Postal 6-641, Mexico DF 06600, Mexico; X.He@cgiar.org (X.H.); Pk.Singh@cgiar.org (P.K.S.)

**Keywords:** Fusarium head blight (FHB), deoxynivalenol (DON), deoxynivalenol-3-*O*-glucoside (D3G), high performance liquid chromatography with tandem mass spectrometry (LC-MS/MS)

## Abstract

Fusarium head blight (FHB) causes significant grain loss and contamination of grains with harmful mycotoxins, especially deoxynivalenol (DON). Fusarium resistance and DON accumulation have been extensively investigated in various cultivars; however, the level of DON-3-*O*-glucoside (D3G) has not been as carefully studied. In this study, we measured accumulated DON and D3G levels in CIMMYT wheat elite germplasm using an analytical method validated in-house. Co-occurring nivalenol (NIV) and ergostrerol (ERG) were also analyzed. LC-MS/MS and LC-UV analyses were applied to the 50 CIMMYT elite wheat lines. D3G showed rather high correlation with DON (*r* = 0.82), while FHB symptoms showed slight correlation with DON and D3G (*r* = 0.36 and 0.32, respectively). D3G/DON ratio varied widely from 8.1 to 37.7%, and the ratio was not related with FHB resistance in this dataset.

## 1. Introduction

Small grain cereals such as wheat and barley are susceptible to pathogenic fungi of the genus *Fusarium*, especially those that cause Fusarium head blight (FHB) or scab. The optimal conditions for fungal infection and propagation are moderate temperature and high humidity. Accordingly, FHB prevails throughout the major wheat-producing areas of the world, with exception of the Indian subcontinent and Australia. Infected spikelets exhibit symptoms of discoloration, and severe FHB can result in shriveled and pale pink grains with low quality and yield [1]. In Japan, there was a historic outbreak of FHB in 1963 in Kyushu district (the southwest part of Japan) following a long period of precipitation that occurred during the anthesis and maturation of the wheat crop. Due to changes in global climate and a wide adoption of conservation agricultural practices, FHB has increased in importance in many countries. For example, in the USA and Canada, FHB was of minimal importance prior to the 1990s, but is now one of the most important wheat diseases [2]. A similar situation arose in China, where the Yellow and Huai wheat production zone (the most important wheat production zone in China) has become increasingly affected with FHB in the last few decades, and this trend will continue in the near future [3].

FHB not only causes economic loss from poor harvest, but is also associated with food safety concerns related to the accumulation of mycotoxins in grains. Yoshizawa identified a major toxigenic compound in scabby kernels, subsequently named deoxynivalenol (DON) [4]. DON contamination is of major concern in the wheat food chain worldwide because the toxin is stable and difficult to remove during food processing [5]. In 2001, the Joint FAO/WHO Expert Committee on Food Additives (JECFA) considered the risk of DON [6]. Many countries have adopted regulatory limits on DON levels (Table 1), and in Asia, nivalenol (NIV) is also considered a threat [7].

A major challenge in assessing the hazard of DON is that the DON content is not always correlated with FHB severity, and contamination can be detected in grains that appear healthy upon visual examination [8]. Another emerging problem is so-called ‘masked’ mycotoxins or mycotoxin glucosides, which are overlooked due to analytical difficulties [9]. The term ‘masked mycotoxin’ was originally applied to *O*-glucosides of zearalenone and DON; however, other mycotoxin derivatives such as *N*-deoxyfructosyl-fumonisins were recently identified, and the term ‘modified mycotoxin’ was proposed [10]. Currently, among mycotoxin glycosides, only deoxynivalenol-3-*O*-glucoside (D3G) is commercially available. 

A combination of various resistance mechanisms may act synergistically to hinder fungal attacks, and several resistance pathways have been identified: resistance to invasion (Type I), resistance to fungal spreading (Type II), resistance to toxin accumulation (Type III), resistance to kernel infection (Type IV), and resistance to yield reduction (Type V) [11]. Among these pathways, Type II has been the most extensively studied, and the other mechanisms, including Type III (resistance to toxin contamination), have also attracted a great deal of attention recently. However, D3G contamination in wheat has been less widely studied in the context of FHB resistance, and no data are available regarding D3G content in CIMMYT wheat germplasm.

**Figure 1:**
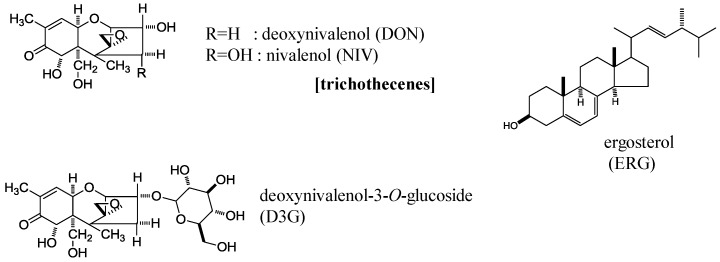
Chemical structures of deoxynivalenol (DON), nivalenol (NIV), deoxynivalenol-3-*O*-glucoside (D3G), and ergosterol (ERG).

In this study, we analyzed NIV, DON, and D3G contents in 50 samples of CIMMYT wheat elite germplasm mainly focused on *Fhb1*, one of the most potent resistant genes [12]. We used a validated analytical method involving liquid chromatography with tandem mass spectrometry (LC-MS/MS) [13] and found the unique property of D3G/DON ratio. Fungal primary metabolite ergosterol (ERG) was also analyzed by a conventional LC-UV method to assess fungal invasion [14] (Figure 1).

## 2. Results

### 2.1. FHB Progression

FHB infection developed well in the field nursery, with the FHB index ranging from 1.5 to 87.2%. The FHB index of the two resistant controls, Sumai#3 and Heilo, was 1.5% and 3.8%, respectively, whereas that of two susceptible controls, Gamenya and Ocoroni, was 63.1% and 87.2%, respectively. The FHB indices of the other 46 accessions ranged from 2.4 to 40.0% (Table S1). The nine *Fhb1*/*Sr2* recombinants all exhibited very low rates of disease, with the FHB index ranging from 2.4 to 3.7% (Table S1). Days to heading (DH) and plant height (PH) were not significantly correlated with FHB index.

### 2.2. Determination of Mycotoxins and ERG

**Figure 2:**
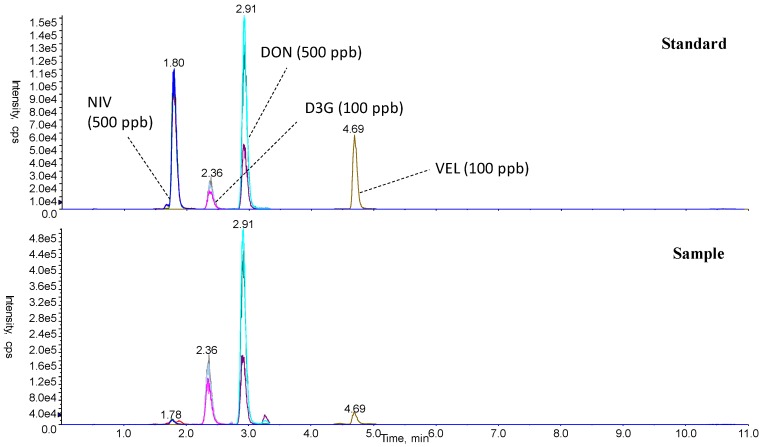
Typical LC-MS/MS chromatograms of a standard NIV, D3G, DON, verrucarol (VEL), and a sample fortified with VEL.

Typical LC-MS/MS chromatograms are shown in Figure 2. NIV, D3G, and DON were simultaneously detected in negative polarity. Using a semi-micro RRHD column and two-step gradient of mobile phase, separation was successfully achieved within 14 min (NIV: 1.7–1.9 min, D3G: 2.3–2.5 min, DON: 2.9–3.0 min, verrucarol (VEL, internal standard): 4.7 min) (Table 2, Figure 2). The values of NIV, D3G, and DON were determined by the average values of duplicate measurements in the same sample, using three sets of (precursor-product) ions to ensure accuracy.

In the 50 CIMMYT wheat germplasms tested, the content of DON was 131–6337 ppb, ranging from low-level contamination (sub-ppm) to high-level contamination (>5 ppm) (Table 3). Sumai#3 contained the lowest level of DON (131 ppb), followed by Heilo (368 ppb) (Table S1); both of these were resistant controls. By contrast, the two susceptible controls (Gamenya and Ocoroni) did not have the highest DON values, and their two values were very different: Gamenya had a value above 5 ppm (5791 ppb), whereas Ocoroni had a value of 1 ppm (1008 ppb). The nine *Fhb1*/*Sr2* recombinants had rather low values of DON (429–1900 ppb), and the majority were below 1 ppm.

All 50 samples contained D3G from 24–2683 ppb (Table 3). Sumai#3 contained the lowest value of D3G (24 ppb), as it did for DON. Heilo contained 149 ppb of D3G, the third lowest value among all samples. The second lowest value (70 ppb) was present in one of the nine *Fhb1*/*Sr2* recombinants (Table S1). The two susceptible controls (Gamenya and Ocoroni) had rather high values of D3G, although they differed substantially from each other (1714 ppb and 396 ppb, respectively).

The D3G/DON ratio was calculated from the absolute values of DON and D3G by the following equation:

D3G/DON ratio = (D3G (ppb)/517.1)/(DON (ppb)/355.1) × 100

D3G/DON ratios ranged from 8.1 to 37.7%, with 10 entries having a ratio less than 20%, 28 within the range of 20–30%, and 12 greater than 30% (Table 3 and Table S1). Sumai#3 had the second lowest value of 12.3%; but the nine *Fhb1*/*Sr2* lines showed a very broad range from 8.1 (the lowest ratio) to 36% (the second highest ratio). The two susceptible controls, Gamenya and Ocoroni, showed moderate ratios of 20.3% and 27%, respectively, whereas the other resistant control Heilo had a high value of 28% (Table S1). 

The CIMMYT wheat germplasm tested also contained NIV at a detectable level, but at most 0.5 ppm (Table 3 and Table S1). Acetylated DONs (15-acetyl-DON and 3-acetyl-DON) were detected in almost all the samples at levels under 90 ppb (data not shown). All germplasm tested contained ERG above 1 ppm. Sumai#3 contained the lowest level of ERG (1.5 ppm), in accordance with its levels of DON and D3G. Heilo contained 3.2 ppb of ERG, the third lowest value. The two susceptible controls had rather high values of ERG (11.9 ppm and 17.6 ppm), but not the highest.

### 2.3. Correlations among Toxin-Related Traits

**Figure 3:**
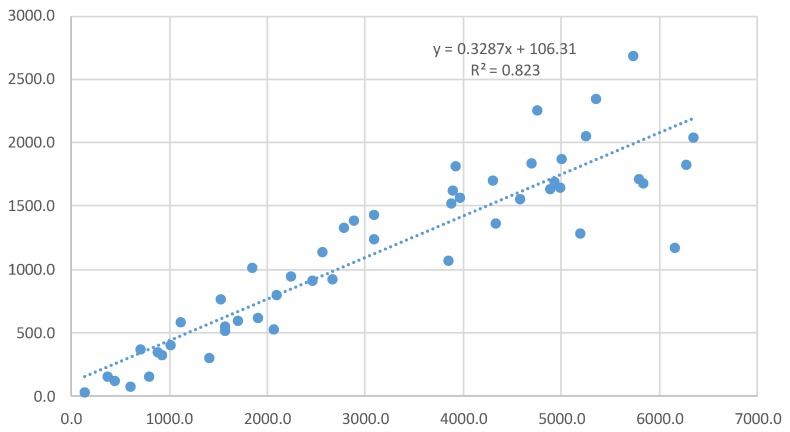
Correlation of D3G (ppb) versus DON (ppb).

**Figure 4:**
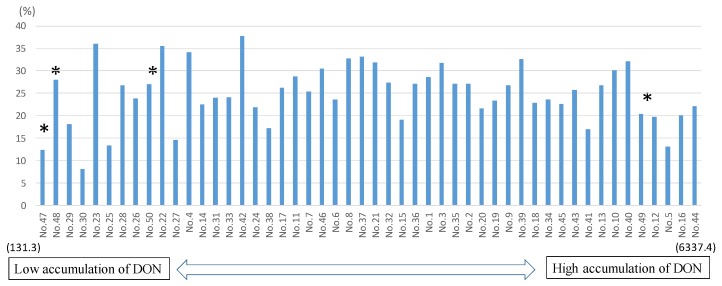
Correlation of D3G/DON ratio versus DON (ppb). * Four controls; No. 47 = SUMAI#3, No. 48 = HEILO, No. 49 = GAMENYA, No. 50 = OCORONI.

The field FHB index was only marginal correlated with DON, D3G, NIV and ERG, with *r* values ranging from 0.32 to 0.37 (Table 4). The highest correlation was between DON and D3G, with an *r* square value of 0.82 (Figure 3), whereas moderate correlations were observed for DON vs. ERG, D3G vs. ERG, NIV vs. D3G, and DON vs. NIV (Table 4). D3G/DON exhibited only a weak correlation with D3G, with an *r* value of 0.32 (Table 4), and was not correlated with DON (Table 4 and Table S1, Figure 4). 

## 3. Discussion

In this study, we investigated the symptoms of FHB at 25 days post-inoculation (dpi), as well as DON and D3G accumulation, in 50 CIMMYT elite wheat breeding lines. A spore suspension mixture of five DON-producing *F. graminearum* strains was used as an inoculum. The FHB index was lowest in two resistant controls (1.5% and 3.8%) and highest in two susceptible controls (63.1% and 87.2%), indicating that the artificial infection experiment worked effectively. ERG, a characteristic fungal primary metabolite, was used as an indicator of fungal biomass. ERG content was over 1 ppm in all tested samples, with the values ranging from 1.5 to 30 ppm, indicating substantial invasion of fungi in all samples. ERG was moderately correlated with DON and D3G, consistent with previous results; however, ERG may not be a good indicator of DON content, as other factors such as Type III resistance are also involved. This is reasonable given that ERG is an essential metabolite for fungal growth, whereas trichothecenes are not.

For the analysis of mycotoxins, DON, NIV, and D3G were analyzed by an LC-MS/MS method, using VEL as an internal standard. Optimization of LC conditions succeeded in shortening the analytical time from 29 min [13] to 13.8 min, with clear separation (Figure 2). DON contents ranged from low (sub-ppm), to medium (1–2 ppm, corresponding to regulatory limits) (Table 1), to high (>5 ppm) levels of contamination, providing an appropriate dataset for this analysis. 

As for the differences among breeding lines, Sumai#3 had the lowest values of FHB index (1.5%), ERG (1.5 ppm), DON (131.3 ppb), and D3G (23.5 ppb). This is reasonable because Sumai#3 is the most famous source of FHB resistance, and is known to carry quantitative trait loci (QTLs) for Type I and Type II resistance [15]. The nine *Fhb1*/*Sr2* recombinants, albeit carrying *Fhb1*, had higher FHB indices and DON contents than Sumai#3, demonstrating that non-*Fhb1* genes in Sumai#3 also contributed to disease reduction and detoxification. 

The development of wheat cultivars resistant to FHB is of global importance. These efforts seek to achieve both FHB resistance and the mitigation of trichothecene mycotoxins such as DON. Lemmens et al. [16] reported the strong detoxification effect of *Fhb1* and speculated that the gene encodes an enzyme responsible for glycosylation, whose homologs identified as UDP-glucosyltransferase in *Arabidopsis* and barley [17,18]. The wheat *Fhb1* gene was recently cloned, although it turned out to be encoding a chimeric lectin rather than a UDP-glucosyltransferase [19], and efforts to clone the detoxification gene in the neighboring region of *Fhb1* is ongoing [20].

The detoxification product, D3G, has attracted increasing attention in light of the fact that it can be converted to DON in the lumen of the human or animal gut, potentially leading to an excess exposure above regulatory limits; nevertheless, D3G is generally regarded as being of much lower toxicity than its precursor DON [21,22]. The D3G/DON ratio has been used as an indicator of DON detoxification activity [21], and is often positively correlated with FHB resistance, as reported in a recent review by Lemmens et al. [22]. Previously, only a few highly resistant wheat varieties were reported to have a D3G/DON ratio higher than 20% [22]; however, among the lines tested in this study, more than half had a ratio higher than 20%, and a few had values higher than 30% (Figure 4 and Table S1). This does not mean, however, that CIMMYT lines with high D3G/DON ratios are highly FHB-resistant, as no significant correlation was found between FHB and D3G/DON in this set of materials. Similarly, Audenaert et al. [23] did not find a significant correlation between the FHB index and D3G/DON ratio in field trials, although they did report a correlation under laboratory conditions. This could be due to either different resistance mechanisms or the complexities of field epidemic environments.

Although it was previously assumed that only *Fhb1* has the ability to detoxify DON into D3G, it now appears that other FHB resistance QTLs also have the ability [23], as demonstrated by our finding that even the highly susceptible varieties had high D3G/DON ratios, and that lines harboring *Fhb1* had a wide range of D3G/DON ratios, from 8.1 to 36.0%. The underlying mechanism of FHB resistance should be investigated in future studies.

## 4. Conclusions

We used LC-MS/MS to analyze DON and D3G contents in 50 CIMMYT wheat elite germplasms with various levels of FHB symptoms. We detected remarkably high D3G/DON ratios even in highly susceptible varieties, which differs from previous findings that higher D3G/DON ratios indicate stronger FHB resistance. This was the first report of the unique D3G/DON feature in CIMMYT germplasm, warranting further studies aimed at understanding the underlying mechanism.

## 5. Materials and Methods

### 5.1. Plant Materials

Fifty CIMMYT elite wheat breeding lines, selected from CIMMYT’s M19^th^FHBSN and C20^th^FHBSN to cover a wide range of FHB resistance, were used in this study. Four controls were included in this set of materials: Sumai#3 and Heilo as resistant controls, and Ocoroni and Gamenya as susceptible controls. Nine *Fhb1*/*Sr2* recombinants described in He et al. [12] were also included to observe the effect of *Fhb1*, whereas all other lines but Sumai#3 do not have the gene.

### 5.2. Chemicals and Standards

A standard solution of D3G was purchased from Wako Chemicals (Osaka, Japan). NIV, DON, and ERG were purchased from Wako, and VEL (an internal standard) was from Sigma-Aldrich Co. (St. Louis, MO, USA). Acetonitrile (LCMS grade) was from Fisher Scientific (Waltham, MA, USA), and distilled water (LCMS grade) was from Kanto Chemical (Tokyo, Japan). Ammonium acetate (chemically pure grade) was from Kanto, and acetic acid (>99.9% of chemically pure grade, not glacial) was from Wako. All other reagents were of analytical grade.

### 5.3. Fungal Strains for Inoculation

*Fusarium* strains are annually collected in late summer from naturally infected wheat spikes from different farms in Mexico. Isolates were first characterized by species- and toxin-specific PCR markers. DON-producing *F. graminearum* strains were evaluated for DON productivity on rice medium, and then tested in a greenhouse for their in vivo aggressiveness. For this experiment, four strains collected in 2015 (Fg15.71, Fg15.73, Fg15.75, and Fg15.76) and one collected in 2014 (Fg14.86) were mixed for field inoculation. For more technical details, see He et al. [24].

### 5.4. Field Trials and FHB Evaluation

The field FHB experiment was conducted at the El Batán station (altitude of 2240 m above sea level, coordinates 19.5° N, 98.8° W, with an average annual precipitation of 625 mm) of CIMMYT, Mexico, during the summer season (May to September) in 2016. Seeds were sown in 1-m double rows and spray-inoculated at each line’s anthesis stage with an inoculum of 55,000 spores/mL; spray inoculation was repeated 2 days later. From the anthesis to early dough stages, the nursery was misted for 10 min per hour from 9 a.m. to 8 p.m. to create a humid environment favorable for FHB development. Wheat/maize rotation and conservation agricultural practices were followed in the nursery to enhance natural inoculum. 

FHB symptoms were evaluated at 25 dpi on the 10 spikes that had been tagged at anthesis. Numbers of infected spikes and symptomatic spikelets of each spike were counted for calculation of the FHB index using the following formula: FHB index = Severity × Incidence [8], where Severity is the averaged percentage of diseased spikelets, and Incidence is the percentage of symptomatic spikes. Plots were sickle-harvested and threshed with a belt thresher set at low wind speed to retain scabby kernels. A sample of 20 g grain of each accession was pulverized in Mexico and sent to Japan for chemical analysis. Additionally, DH and PH were scored for these materials in order to test their potential association with the FHB index.

### 5.5. Preparation of Stock and Working Solutions for Chemical Analysis

Mycotoxin solutions for stock and calibration were prepared at NARO as described below. NIV, DON, and VEL obtained in the crystalline form were accurately weighed and individually dissolved in acetonitrile. Solvent volumes were adjusted to yield concentrations of 100–200 mg/L. D3G solution in acetonitrile was used as the stock at a concentration of 50 mg/L. These stock solutions were stored in amber glass containers at 4 °C (NIV, D3G, and DON) or at −20 °C (VEL) to prevent photo-degradation and evaporation of the mycotoxins. An internal standard solution (VEL at the concentration of 2 mg/L) was prepared by diluting the stock solution in acetonitrile, and stored in amber glass containers at −20 °C. For working solutions, each stock solution was dried under a stream of nitrogen gas and re-dissolved by dilution in acetonitrile/water/acetic acid (5/94/1, *v*/*v*/*v*) as described in a previous work [13]. ERG working solutions (2.5–20.0 mg/L ethanol) were used as calibration samples. All working solutions were stored at 4 °C.

### 5.6. Extraction and Purification of Mycotoxins and ERG

Extraction and purification of mycotoxins were performed as described in a previous work by Nakagawa et al. [13]. Each ground sample (10 g) was placed in glassware and fortified with 0.5 mL of internal standard solution (2 mg/L VEL in acetonitrile) to an adjusted concentration of 0.1 mg/kg. After being kept in the freezer or refrigerator for more than 12 h, the fortified samples were incubated at room temperature for approximately 30 min. Next, 40 mL of acetonitrile/water (80/20, *v*/*v*) and 0.4 mL of acetic acid (>99.9%) were added, and the mixture was homogenized for 5 min or vigorously shaken for 30 min. The resultant slurry was centrifuged at 2000× *g* for 10 min, and a portion of the supernatant (15 mL) was loaded onto a solid-phase Presep C18 extraction (SPE) column (ODS) (2 g/15 mL) (Wako, Part No. 296-34091). The resulting eluate was consecutively loaded on a multifunctional Bond Elut Mycotoxin column (Agilent Technologies, Santa Clara, CA, USA). After discarding the initial 3 mL of the solvent coming off the column, a 1.6 mL aliquot was removed from the following eluent and dried under a nitrogen gas stream at 40 °C. The residue was re-dissolved in 0.4 mL of acetonitrile/water/acetic acid (5/94/1, *v*/*v*/*v*) and filtered with a DISMIC-13HP hydrophilic PTFE disposable syringe filter unit (pore size, 0.20 μm) (Toyo Roshi Kaisha, Ltd., Tokyo, Japan), and the resultant filtrate was subjected to LC-MS/MS analysis. 

ERG extraction, saponification, and purification by liquid–liquid partitioning were conducted by the methods described previously [14] with some modifications. Briefly, 1.0 g of sample was extracted with 5.0 mL of methanol in a closed vessel. Exactly 0.6 mL of supernatant was placed in a 2-mL tube, to which 0.25–0.3 g of potassium hydroxide was added; the sample was then warmed at 65 °C for 15 min. n-Hexane (0.3 mL) was added, the tube was closed tightly, and the sample was incubated at 65 °C for 30 min. After the sample had cooled to room temperature, 0.15 mL of distilled water and 0.6 mL n-hexane were added, and the mixture was then shaken gently. The hexane layer was transferred to a glass tube, and the extraction was repeated another two times. The combined hexane layers were evaporated to dryness, and the residue was re-dissolved in 0.12 mL of ethanol to serve as a sample for HPLC analysis.

### 5.7. Analysis of Mycotoxins and ERG

Detection and quantification of mycotoxins was performed with a 4000QTRAP LC-MS/MS system (Sciex, Foster City, CA, USA) equipped with a 1290 Series HPLC system (Agilent Technologies, Santa Clara, CA, USA). Chromatographic separation was performed at 40 °C on a ZORBAX Eclipse XDB-C18 RRHD Solvent Savor column (150 mm × 2.1 mm i.d., 1.9 μm particle size) (Agilent). Eluents consisted of water/acetic acid (99.9:0.1, *v*/*v*) containing 0.5 mM ammonium acetate (eluent A), and acetonitrile/acetic acid (99.9:0.1, *v*/*v*) (eluent B), with stepwise gradient in 13.8 min (0–1 min: hold 12% B; 1–6.5 min: from 12 to 68% B; 6.5–9 min: from 68 to 85% B; 9–10 min: hold 85% B; 10–10.1 min: from 85 to 12% B; 10.1–13.8 min: hold 12% B to equilibrate). Sample injection was conducted at a volume of 3 µL. Ionization was conducted with an electro spray ionization (ESI) probe in negative polarity. Data acquisition was performed in the multiple reaction monitoring (MRM) mode of LC-MS/MS, and the monitor ions used for detection of the respective mycotoxins are shown in Table 2. 

Conventional HPLC–UV was performed for ERG analysis. HPLC separation was performed on a reversed-phase C18 column CAPCELL PAK, UG120 (250 mm × 3.0 mm i.d., 5 µm particle size) (Shiseido, Tokyo, Japan). Mobile phase was 98% methanol/acetonitrile (1:1) mixed with 2% HPLC grade water. The column heater was set at 30 °C, and the injection volume was 10 µL. ERG was detected by UV absorption at a wavelength of 282 nm.

## Figures and Tables

**Table 1:** Distribution of regulatory limits for deoxynivalenol (DON) in wheat (flour) or cereals in different countries.

ppb	Number of Countries
1100–2000	4
1000	9
750	19
300–700	5

**Table 2:** LC-MS/MS conditions for the detection of NIV, D3G, and DON.

Analyte	Precursor Ion	Product Ion	Retention Time (min)
NIV (C_15_H_20_O_7_)	371.1 [C_15_H_20_O_7_+CH_3_COO]^−^	280.8	1.74–1.81
		310.7	1.71–1.87
		59.0	1.74–1.87
D3G (C_21_H_30_O_11_)	517.1 [C_21_H_30_O_11_+CH_3_COO]^−^	456.9	2.28–2.48
		426.9	2.29–2.49
		58.9	2.28–2.48
DON (C_15_H_20_O_6_)	355.1 [C_15_H_20_O_6_+CH_3_COO]^−^	294.8	2.86–3.00
		264.8	2.86–3.00
		58.9	2.86–3.00

**Table 3:** Distribution of FHB index, ERG, NIV, D3G, DON, and D3G/DON ratio among 50 CIMMYT wheat germplasm samples.

	FHB Index	ERG (ppm)	NIV (ppb)	D3G (ppb)	DON (ppb)	D3G/DON Ratio (%)
Range	1.48–87.22	1.49–29.57	3.7–496.5	23.5–2682.6	131.3–6337.4	8.1–37.7
Average	19.03	8.99	96.8	1145	3161	24.9

**Table 4:** Correlation coefficients (*r*) among FHB index and individual toxin related traits evaluated in this study.

	FHB	ERG	D3G	DON	D3G/DON	NIV
FHB	1					
ERG	0.37 **	1				
D3G	0.32 *	0.55 ***	1			
DON	0.36 **	0.55 ***	0.91 ***	1		
D3G/DON	0.03	0.09	0.32 *	−0.01	1	
NIV	0.35 *	0.25	0.45 ***	0.42 **	0.17	1

FHB, FHB index; ERG, ergosterol; D3G, deoxynivalenol-3-*O*-glucoside; DON, deoxynivalenol; D3G/DON, the molar ratio between D3G and DON; NIV, nivalenol statistical significance: * *p* < 0.05, ** *p* < 0.01, *** *p* < 0.001.

## Supplementary Materials

The following are available online at www.mdpi.com/2072-6651/9/8/238/s1, Table S1: Whole dataset of 50 lines.

## Abbreviations

FHB—Fusarium head blight; DON—deoxynivalenol; NIV—nivalenol; D3G—deoxynivalenol-3-*O*-glucoside; LC-MS/MS—liquid chromatography with tandem mass spectrometry; ERG—ergosterol; VEL—verrucarol; dpi—days post-inoculation; DH—days to heading; PH—plant height; QTL—quantitative trait locus.
